# Development of a real-time PCR method for rapid diagnosis of canine babesiosis and anaplasmosis

**DOI:** 10.1186/s13071-021-04756-9

**Published:** 2021-05-20

**Authors:** Agnija Kivrane, Agne Namina, Maija Seleznova, Sarmite Akopjana, Valentina Capligina, Renate Ranka

**Affiliations:** Latvian Biomedical Research and Study Centre, Ratsupites Street 1, Riga, Latvia

**Keywords:** *Babesia canis*, *Anaplasma phagocytophilum*, Genotyping, *Bc28.1* gene, *Mdh* gene, Real-time PCR

## Abstract

**Background:**

Canine babesiosis and anaplasmosis, caused by *Babesia canis* and *Anaplasma phagocytophilum*, respectively, are significant tick-borne diseases in Baltic countries. Both diseases can be diagnosed on the basis of clinicopathological findings, by direct pathogen detection in blood smears or by indirect pathogen detection; however, because of high selectivity and specificity, molecular methods may be advantageous. The goal of this study was to develop a duplex real-time polymerase chain reaction (RT-PCR) method for the detection of *B. canis* and *A. phagocytophilum* in canine clinical samples.

**Methods:**

Sequence-based polymorphism analysis of genes encoding *B. canis*-specific merozoite surface protein Bc28.1 (*Bc28.1*) and *A. phagocytophilum* malate dehydrogenase (*mdh*) was performed on pathogen isolates present in Latvian domestic dogs. The obtained results were used to design a species-specific duplex RT-PCR assay.

**Results:**

The presence of three *B. canis Bc28.1* gene sequence types was revealed in canine samples with a nonuniform geographical distribution, and two types of *A. phagocytophilum mdh* genes were detected. The novel duplex RT-PCR assay provided correct classification of samples positive and negative for *B. canis* and *A. phagocytophilum*. The analytical sensitivity of this assay was ten gene copies/ reaction for both pathogens.

**Conclusions:**

A novel duplex RT-PCR molecular method was developed for the detection of *B. canis* and *A. phagocytophilum* in canine clinical samples. Sequence variability of *Bc28.1* and *mdh* genes indicated the genetic variability of *B. canis* and *A. phagocytophilum* isolates occurring in Latvian domestic dogs.

**Graphic Abstract:**

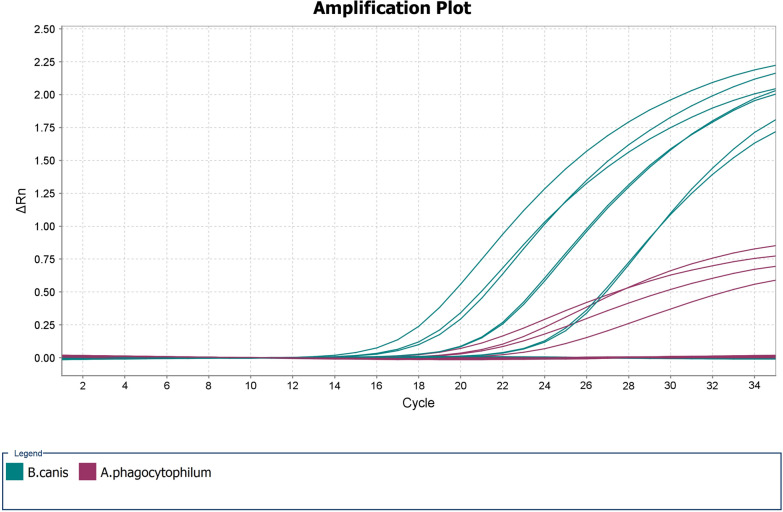

**Supplementary Information:**

The online version contains supplementary material available at 10.1186/s13071-021-04756-9.

## Background

Canine babesiosis is an important tick-borne disease caused by species of the genus *Babesia* [1]. Clinical manifestations vary, and the disease can be lethal if left untreated [2]. In the last decade, canine babesiosis has been established as an endemic disease with a seasonal pattern in the Baltic countries of Lithuania and Latvia and is associated with the spread of the tick vector *Dermacentor reticulatus* [3,6]. Molecular testing has shown that *Babesia canis* was the sole causative agent of the disease in published canine babesiosis case reports from Latvia, Lithuania and Estonia [5, 7,9]. Another tick-borne pathogen of veterinary significance is the gram-negative intracellular bacterium *Anaplasma phagocytophilum*, which is transmitted mainly by *Ixodes* spp. ticks and has important zoonotic potential [10]. Studies performed in Europe showed that 3 to 57% of dogs were exposed to *A. phagocytophilum* [11]. In Latvia, the reported seroprevalence of anaplasmosis in dogs is 1117% [12]. In a recent large-scale countrywide study, *A. phagocytophilum* was identified at similar proportions in field-collected Latvian *I. ricinus*, *I. persulcatus* and *Dermacentor reticulatus* ticks, with an overall prevalence of 1.09% [13].

Rapid diagnosis and differentiation of both infections is highly important for timely initiation of effective treatment [14]. The direct blood smear detection method is not sensitive enough to detect either *A. phagocytophilum* or *B. canis*, while serological testing is not informative because it is not suitable in the acute phase of the infection, does not distinguish between ongoing disease and previous exposure, and cross-reactivity could interfere with the specificity of the test [2, 11, 14]. Molecular testing has been shown to be appropriate and advantageous because of improved diagnostic specificity and sensitivity [1, 11]. For molecular detection of *Babesia* spp., numerous genus- and species-specific polymerase chain reaction (PCR)-based methods have been reported that target various genes, including *18S* ribosomal RNA, cytochrome c oxidase 1 (*COX1*) and internal transcribed spacers (*ITS*) [15,19]. Molecular assays for *A. phagocytophilum* are mostly based on the amplification of the *16S* ribosomal RNA and *groESL* heat shock operon genes [20,22]. Real-time polymerase chain reaction (RT-PCR) methods were highlighted as more convenient and time-saving for clinical applications, and several singleplex and multiplex RT-PCR assays for detecting *Babesia* spp. and *A. phagocytophilum* in clinical samples have been reported; however, none of them were tested for simultaneous detection and differentiation of *B. canis* and *A. phagocytophilum* infections in clinical samples [23,28]. Both canine babesiosis and anaplasmosis are present in Latvia and elsewhere in Europe; in addition, the risk of coinfection exists because of the sympatric occurrence of the principal vectors of canine babesiosis and anaplasmosis, *I. ricinus* and *D. reticulatus* [13].

In the present study, sequence-based polymorphism analysis of genes encoding *B. canis*-specific merozoite surface protein Bc28.1 (*Bc28.1*) and *A. phagocytophilum* malate dehydrogenase (*mdh*) was performed. The obtained results were used to design a species-specific duplex RT-PCR assay for the rapid detection and discrimination of *B. canis* and *A. phagocytophilum* in dogs.

## Methods

### Samples

The blood samples from dogs used in this study were collected by Latvian veterinarians. The first sample set comprised 262 clinical samples submitted during the period from 2016 to 2019 [5]. Five samples were excluded because of insufficient amounts of DNA. The second clinical sample set (*n*=94 samples) was submitted by veterinarians for molecular testing from 2011 to 2014.

DNA isolation from clinical samples was performed as described previously by Berzina et al. [12]. All clinical samples were screened by seminested PCR targeting the *18S* rRNA gene fragment for *Babesia* spp. [17] and species-specific nested PCR targeting *16S* rRNA gene fragments for *A. phagocytophilum* [22]. *Babesia spp.* infection was confirmed, and pathogens were identified by sequencing the amplification products. As a negative control, PCR mixtures without DNA were used. As positive controls for PCR, the following specimens were used: *A. phagocytophilum* Webster strain (kindly donated by Friederike von Loewenich, Institute of Medical Microbiology, University of Freiburg, Germany) and *B. canis*-positive clinical sample Lv-dog 2 (positive DNA sample from dog blood), which was obtained in a previous study [8].

*B. canis*- and *A. phagocytophilum*-positive clinical samples were further used for *Bc28.1* and *mdh* gene polymorphism analysis, respectively. Both clinical sample sets were used for the specificity assessment of the novel RT-PCR method. *Theileria annae* (*B. vulpes*)-positive DNA samples were kindly donated by Prof. Andrei D. Mihalca, University of Agricultural Sciences and Veterinary Medicine of Cluj-Napoca, Romania. *Ehrlichia canis*- and *A. platys*-positive DNA samples were kindly donated by Assoc. Prof. Hui-Wen Chen, National Taiwan University, Taiwan.

### PCR amplification and sequencing of *B. canis Bc28.1* and *A. phagocytophilum mdh* genes

The full-length *Bc28.1* gene of *B. canis* was amplified using PCR in *B. canis*-positive clinical samples according to the protocol by Carcy et al. [29] with a few minor modifications. The oligonucleotide primer pair F5UTR281&2/R3UTR281 was used for amplification of the 852-nt-long *Bc28.1* gene fragment (Table 1) [29]. For each PCR, 26l of the reaction mixture was prepared, which consisted of 1reaction buffer BD with (NH_4_)_2_SO_4_ and Tris-HCl, 2.5mM MgCl_2_, 0.2mM for each dNTP, 0.2M for each primer, 0.8 U of FIREPol DNA polymerase and 2l of target DNA. Amplification was performed at the following temperatures: initial denaturation at 94C for 3min, followed by 35 amplification cycles (94C for 30s, 55C for 30s, 72C 60s), and a final extension step at 72C for 7min.

**Table 1:** Primers and probes used in this study

Target gene	Primer/probe name	Sequence (53)	Product size	References
*B.canis Bc28.1*	F5UTR281&2	AGTCGATACCTCCGAGAATAG	852 nt	[29]
	R3UTR281	CATTACGCCCACAAA TAGTCA		[29]
*A. phagocytophilum mdh*	mdh 1fa	GTGTTGCGGGTATCTGTCA	593 nt	[30]
	mdh 2ra	TCCTCCCTTGCGAGTCCT		[30]
*B.canis Bc28*	BC28F3	GCTACGTCCGTTGAAGCC	65 nt	This study
	BC28R3	TCAGCGGAATAACGTTCAGC		This study
	BC28	FamAGCCAGTCGATCTGCTCCTTTAAGCT		This study
*A. phagocytophilum mdh*	APmdhF	CAGACTATGCAGCTATTGAGGG	100 nt	This study
	APmdhR	GCATTAGCCATGAGCAAATCTTC		This study
	APmdhProbe	HexTCCTTTCTAGGAAGGCCTGC		This study

The 593-bp fragment of *mdh* gene of *A. phagocytophilum*, which is a highly conserved housekeeping gene used for multilocus sequence typing (MLST) analysis, was amplified using the PCR primers mdh 1fa and mdh 2ra according to the MLST database (http://pubmlst.org/aphagocytophilum) (Table 1) [30]. For each PCR, 26l of the reaction mixture was prepared, which consisted of 1Phusion HF Buffer (with 7.5mM MgCl_2_), 0.2mM for each dNTP, 0.3M for each primer, 0.4 U of Phusion Hot Start II High-Fidelity DNA Polymerase and 2l of target DNA. Amplification was performed at the following temperatures: initial denaturation at 98C for 3min, followed by 40 amplification cycles (98C for 20s, 65C for 30s, 72C 60s) and a final extension step at 72C for 5min.

All PCRs were performed by using Mastercycler epgradient S (Eppendorf AG, Hamburg, Germany). Negative PCR controls (PCR mixtures without DNA) were included in each run. The obtained PCR products were analyzed by agarose gel electrophoresis in a 1.5% agarose gel with 0.2g of ethidium bromide/ml. If necessary, amplification products were stored at 20C until further analysis.

BrilliantDye Terminator Cycle Sequencing Kit v1.1 (NimaGen, Nijmegen, Netherlands) was used to sequence the amplification products. Sequencing was carried out according to a standard protocol using an ABI Prism 3100 Genetic Analyzer (PerkinElmer, Waltham, MA, USA). The MEGA and FinchTV programs and ClustalW alignment algorithm [31] were used for sequence alignment and analysis; Basic Logical Alignment Search Tool (BLAST, https://blast.ncbi.nlm.nih.gov/Blast.cgi) was used for sequence comparison to previously published data in GenBank. The obtained *B. canis Bc28.1* gene sequence types were submitted to GenBank with the following accession numbers: MN832760, MN832761 and MN832762. The obtained *A. phagocytophilum mdh* gene sequences were submitted to GenBank with the following accession numbers: MW822581, MW822582, MW822583, MW822584, MW822585 and MW822586.

### Phylogenetic analysis of *B. canis Bc28.1* and *A. phagocytophilum mdh* genes

*B. canis Bc28.1* gene sequence types were distinguished on the basis of nucleotide substitutions according to a previously reported classification [30]. All available *B. canis Bc28.1* gene sequences were retrieved from GenBank (*n*=18; assessed on 1 September 2020). Phylogenetic analysis was performed using 57 nucleotide sequences, including 39 sequences from this study; the heterogeneous isolate was omitted. *A. phagocytophilum mdh* gene alleles were distinguished on the basis of nucleotide substitutions according to the *A. phagocytophilum* MLST database [30]. All available *A. phagocytophilum mdh* sequences were retrieved from the MLST database (520 isolates and 42 alleles; database was accessed on 1 September 2020). Identical sequences from the same country were merged, and detailed information about the total number and origin of these samples was indicated next to the branch in the phylogenetic tree. The final phylogenetic analysis involved 79 nucleotide sequences, including 6 sequences from this study. The most suitable evolutionary model was determined in MEGA based on the lowest (Bayesian information criterion, BIC) scores. The evolutionary history for the *B. canis Bc28.1* gene was inferred by using the maximum likelihood method based on the Jukes-Cantor model [32]. The evolutionary history for *A. phagocytophilum mdh* gene was inferred by using the maximum likelihood method based on the Hasegawa-Kishino-Yano model [33]. Phylogenetic analysis was conducted in MEGA7 [34]. The phylogenetic trees were midpoint rooted.

### Preparation of plasmid standard controls by molecular cloning

The full-length *B. canis Bc28.1* gene and partial-length *A. phagocytophilum mdh* gene were amplified by PCR using the F5UTR281&2/R3UTR281 primer pair and mdh 1fa/mdh 2ra primer pair, respectively (Table 1). For each PCR, 25l of the reaction mixture was prepared, which consisted of 1Phusion HF Buffer with MgCl_2_, 0.2mM for each dNTP, 0.3M for each primer, 0.4 U of Phusion Hot Start II High Fidelity DNA Polymerase and 2 l of target DNA. Amplification was performed at the following temperatures: initial denaturation at 98C for 30s, followed by 35 amplification cycles (98C for 10s, 62C (*mdh* gene)/60C (*Bc28.1* gene) for 30s, 72C 30s), and a final extension step at 72C for 5min. The obtained amplification products were analyzed by agarose gel electrophoresis. If necessary, amplification products were stored at 20C until further analysis.

The PCR products were cloned into the pJET1.2/blunt cloning vector using the CloneJET PCR Cloning Kit (Thermo Fisher Scientific, USA) according to the manufacturer's instructions. Plasmids with confirmed *Bc28.1* and *mdh* gene inserts were purified from bacterial cultures. DNA concentration and purity were assessed using a NanoDrop spectrometer (Thermo Fisher Scientific, USA), and DNA copy number was calculated using a Thermo Fisher Scientific DNA Copy Number and Dilution Calculator (https://www.thermofisher.com/lv/en/home/brands/thermoscientific/molecularbiology/molecularbiologylearningcenter/molecularbiologyresourcelibrary/thermoscientificwebtools/dnacopynumbercalculator.html).

Recombined plasmids were spiked with uninfected canine gDNA (~30ng/l) and used as positive controls and for validation of the RT-PCR assay.

### Design of species-specific duplex RT-PCR assay

Primers and probes were designed using the online-based Primer-BLAST tool (https://www.ncbi.nlm.nih.gov/tools/primerblast/). All primers and probes were inspected for hairpin and dimer formation. To achieve optimal performance of the assay, various primer and probe concentrations were tested (data not shown).

For the *B. canis* assay, primers and probes were created on the basis of sequence alignment, which comprised *Bc28.1* gene sequences from this study and the GenBank database. The primer pair BCF3/BCR3 and BC28 probes were designed to amplify 65-bp-long gene fragments, which did not involve any polymorphic sites (Table 1). For the *A. phagocytophilum* assay design, sequences of *mdh* gene alleles (*n*=42) from the *A. phagocytophilum* MLST database were exported and aligned. Primers APmdhF/APmdhR and probe APmdhP were designed to amplify 100-bp-long gene fragments at the most conserved part of the gene (Table 1).

Primers and probes were synthesized by Metabion International AG, Germany. The RT-PCR mixture consisted of 1TaqMan Fast Advanced Master Mix (Thermo Fisher Scientific, USA), 0.4M Hex-labeled APmdhP probe, 0.5M APmdhF/APmdhR primers, 0.25M Fam-labeled BC28 probe and BCF3/BCR3 primers, 25l of target DNA and nuclease-free water to a final volume of 20 l. The thermocycler conditions were adjusted considering the manufacturers recommendations, and the primer and probe melting temperatures were calculated. To prevent contamination, uracil-DNA glycosylase (UNG) was added to the reaction mixtures. Amplification was performed at the following temperatures: UNG pretreatment at 50C to avoid possible reamplification of carryover amplification products and initial denaturation at 95C for 20s, followed by 35 amplification cycles (95C for 1s, 60C for 20s, data acquisition). Plasmid standard controls and negative controls were included in each run. For RT-PCR assays and data collection and analysis, QuantStudio 7 Flex Real-Time PCR System and QuantStudio Real-Time PCR Software v1.3. (Thermo Fisher Scientific, USA) were used.

### Validation of species-specific duplex RT-PCR assay

The linearity and efficiency () of the novel RT-PCR method were defined using serial tenfold dilutions of standard controls with concentrations ranging from 80 to 8,000,000 gene copies per reaction. Serial dilutions consisted of plasmid controls diluted in uninfected canine gDNA (~30ng/ul). Each dilution contained both target DNA standards. Three runs under repeatable conditions were performed. Standard curves for both pathogens were constructed by plotting the threshold cycle (C_t_) versus target gene copies per reaction. To prevent contamination, UNG was added to the reaction mixture. Negative controls were included in each run. Linear regression analysis was performed by QuantStudio Real-Time PCR Software. The amplification efficiency () was calculated according to the formula: =10010^1/slope^1.

Analytical sensitivity was characterized using the lower limit of detection (LOD) or lowest concentration of target DNA at which 95% of samples were detected as positive. To determine the LOD at the nonlinear range of the assay, 5 and 10 gene copies/ reaction in canine gDNA (~30ng/ul) were chosen. Each dilution contained both target DNA standards. Twenty intra-assay replicates for each concentration under repeatable conditions were analyzed. Negative controls were included in each run.

Clinical specificity was evaluated by analyzing two independent and characterized clinical sample sets (*n*=257 and *n*=94) that included *B. canis*- and *A. phagocytophilum*-positive and -negative clinical samples. The obtained RT-PCR results were compared to the PCR results mentioned before. The possible cross-reactivity of the assay was assessed by using *B. vulpes* (*n*=8)-, *E. canis* (*n*=3)- and *A. platys* (*n*=3)-positive samples. Additionally, for PCR product size confirmation, 20 randomly chosen RT-PCR amplification products of clinical samples were analyzed by 2.0% agarose gel electrophoresis (data not shown).

## Results

### Molecular analysis of sample sets

For the first sample set, molecular screening revealed 40 *B. canis*- and 5 *A. phagocytophilum*-positive clinical samples, and 212 samples were characterized as uninfected. The second clinical sample set contained two *B. canis* and one *A. phagocytophilum*-positive clinical samples and 91 uninfected samples.

### Polymorphism analysis of the *B. canis Bc28.1* gene

**Fig. 1:**
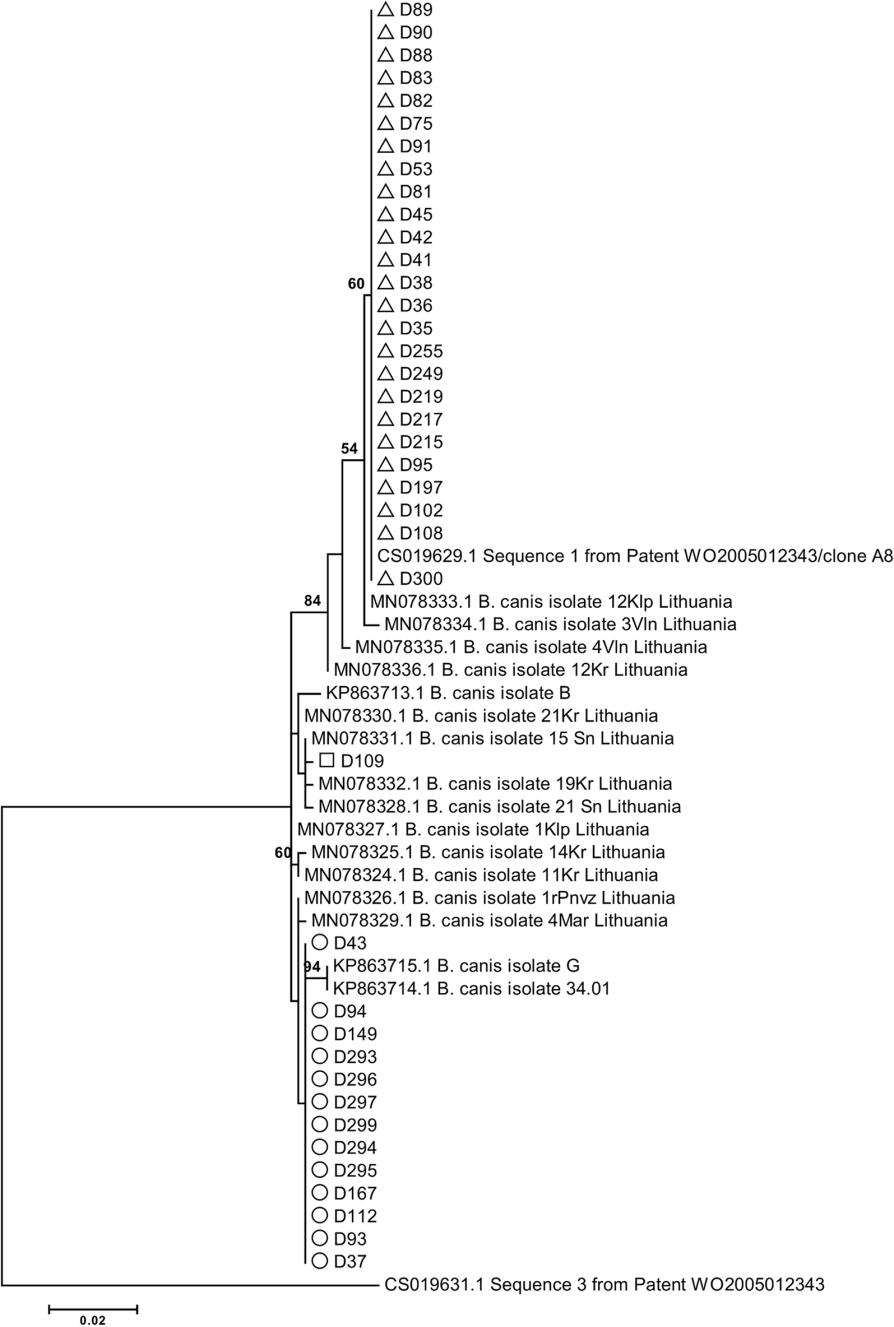
Molecular phylogenetic analysis of *B. canis Bc28.1* gene sequences. The evolutionary history was inferred by using the maximum likelihood method based on the Jukes-Cantor model. The tree with the highest log likelihood (1361.7900) is shown. The percentage of trees in which the associated taxa clustered together is shown next to the branches. The tree is drawn to scale, with branch lengths measured in the number of substitutions per site. All positions containing gaps, and missing data were eliminated. There were a total of 629 positions in the final dataset. Bootstrap values < 50% were omitted. *B. canis* isolates from this study were indicated by white triangles (isolate D197 type), white circles (isolate D93 type) and white square (isolate D109)

Full-length *Bc28.1* gene sequences were obtained from 40 *B. canis-*positive clinical samples. Analysis of the *Bc28.1* gene sequences revealed 24 polymorphic sites that allowed the classification of *B. canis* isolates into three groups corresponding to three sequence types: D197, D93 and D109 (group names were chosen according to the representative samples) (Table 2; Fig.1). The *Bc28.1* sequence type detected in the *B. canis* isolate D197 was identical to the A8 genotype previously reported by Carcy and colleagues [29]; in total, this sequence type was detected in 25 (62.5%) samples. The *Bc28.1* sequence type of the *B. canis* isolate D93 was closely related but not identical (three nucleotide difference) to the 34.01 genotype previously reported by Carcy and colleagues [29] (Table 2; Fig.1); it was detected in 13 (32.5%) samples; 1 *B. canis* sample was heterozygous. The *Bc28.1* gene sequence variant of the D109 sample has not been reported previously; in phylogenetic analysis, it clustered together with three *B. canis* isolates from Lithuania (isolates 19Kr, 15Sn and 21Sn) (Fig.1).

**Table 2:** Polymorphism analysis of *B. canis Bc28.1* gene in canine babesiosis samples

Genotype	Nucleotide positions^b^													
	26	35	36	38	129	242	248	259	314	316	412	493	494	505
Isolate A8 (CS019629.1)^a^	G	C	T	T	G	G	A	G	G	A	C	A	G	A
Isolate D197	G	C	T	T	G	G	A	G	G	A	C	A	G	A
Isolate 34.01 (KP863714.1)^a^	A	T	C	C	C	A	C	A	G	G	T	C	A	T
Isolate D93	A	T	C	C	C	G	C	G	G	G	T	C	A	T
Isolate D109	A	T	T	C	G	G	C	G	A	A	T	C	A	T
Isolate B (KP863713.1)^a^	A	T	T	C	G	G	C	G	G	A	T	C	A	T
	586	599	634	643	684	692	693	694	739	815				
Isolate A8 (CS019629.1)^a^	C	C	A	T	C	G	A	G	C	G				
Isolate D197	C	C	A	T	C	G	A	G	C	G				
Isolate 34.01 (KP863714.1)^a^	A	G	T	C	A	del	del	del	A	C				
Isolate D93	A	C	T	C	A	del	del	del	A	C				
Isolate D109	A	C	T	C	A	del	del	del	C	G				
Isolate B (KP863713.1)^a^	A	C	T	C	C	G	A	G	A	C				

^a^Isolates described in Carcy et al. 2015 [19]

^b^Nucleotide positions according to the reference sequences CS019629.1 (*Bc28.1* gene)

**Fig. 2:**
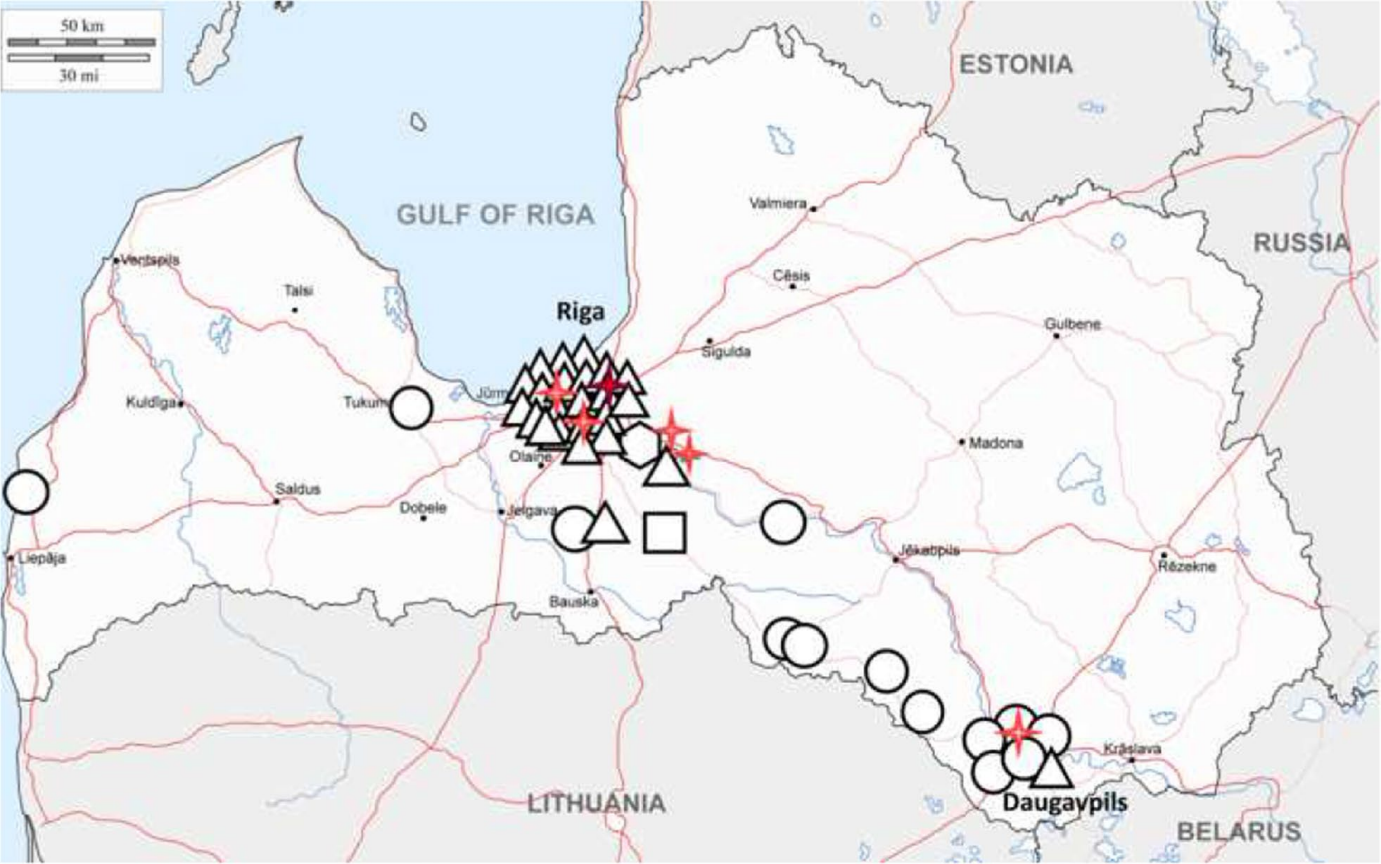
Geographical distribution of *B. canis Bc28.1* gene and *A. phagocytophilum mdh* gene sequence types in Latvia. Triangle: *B. canis* isolate D197 type; circle: *B. canis* isolate D93 type; hexagone: *B. canis* heterogenic sample; rectangle: *B. canis* isolate D109 type. Four-point red star: *A. phagocytophilum mdh* allele 2; four-point dark red star: *A. phagocytophilum mdh* allele 3

The geographical distribution of *B. canis Bc28.1* sequence types in Latvia was further evaluated. The results showed that the *B. canis* A8/D197 type was mainly detected in Riga and the Riga regions (21 of 25 samples), while the 34.01-like/D93 type was detected in different regions of the country; most often, this sequence type was observed in the Daugavpils region (Fig.2). *B. canis* sample D109 was obtained near the Lithuanian border.

### Phylogenetic analysis of the *A. phagocytophilum mdh* gene

**Fig. 3:**
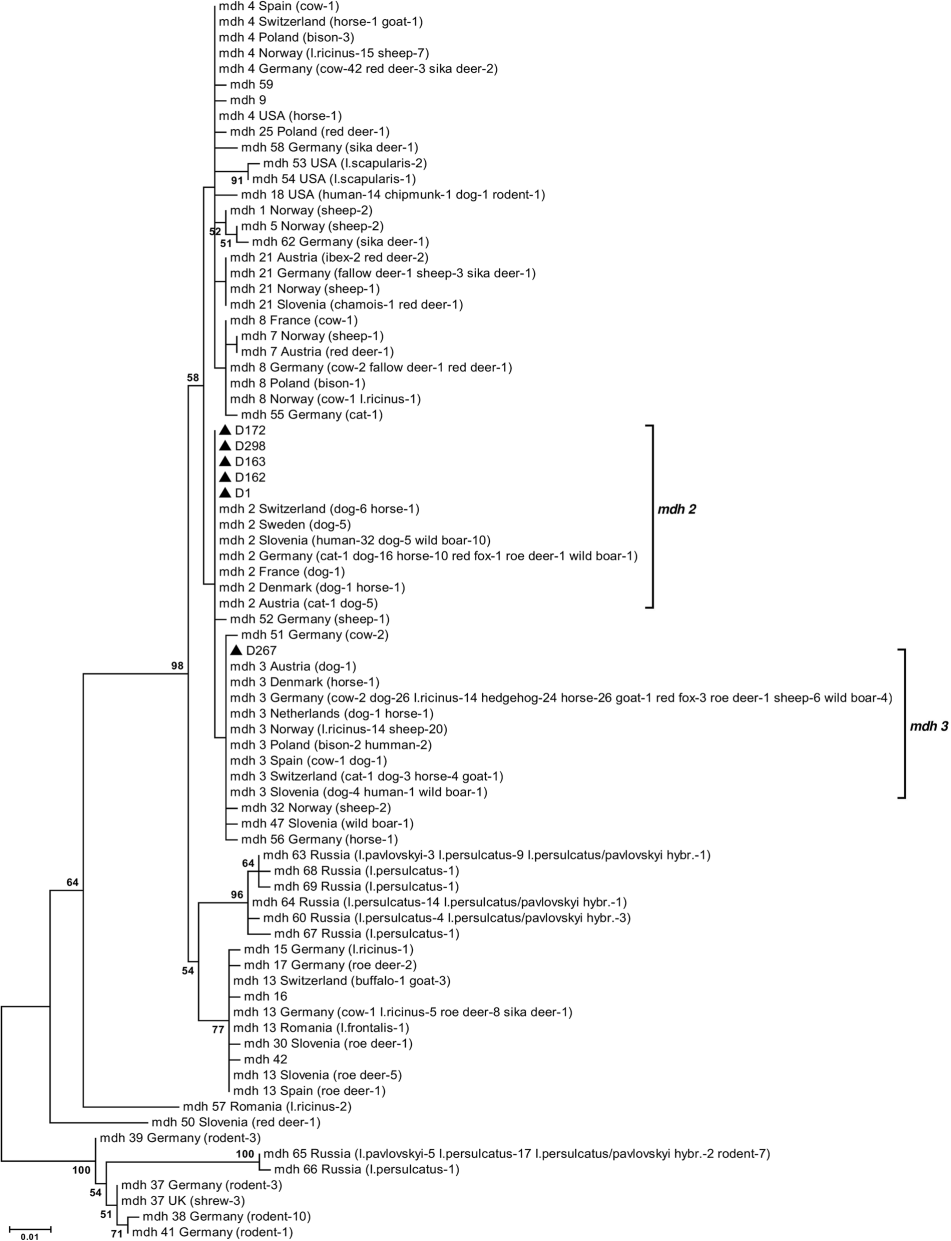
Molecular phylogenetic analysis of *A. phagocytophilum mdh* gene sequences. The evolutionary history was inferred using the maximum likelihood method based on the Hasegawa-Kishino-Yano model. The tree with the highest log likelihood (1159.3952) is shown. The percentage of trees in which the associated taxa clustered together is shown next to the branches. The tree is drawn to scale, with branch lengths measured in the number of substitutions per site. All positions containing gaps and missing data were eliminated. There were a total of 387 positions in the final dataset. Bootstrap values < 50% were omitted. *A. phagocytophilum* isolates from this study were indicated by black triangles

Partial-length (593bp) *mdh* gene sequences were obtained from six *A. phagocytophilum*-positive clinical samples. Sequence-based phylogenetic analysis revealed that five *A. phagocytophilum* isolates from Latvian dogs carried the MLST-based *mdh* gene allele 2 and one sample carried the *mdh* gene allele 3; both allele clusters contained numerous *A. phagocytophilum* isolates from Europe (Fig.3). All but one *A. phagocytophilum*-positive canine clinical sample, including that of the *mdh* gene allele 3 cluster, was obtained in the Riga region; one sample was obtained in Daugavpils (Fig.2).

### Linearity (R^2^) and efficiency () of the novel duplex RT-PCR assay

*B. canis Bc28.1* sequences obtained in this study along with *A. phagocytophilum mdh* gene sequences were further used for novel species-specific duplex RT-PCR assay design. During the validation step, linear regression analysis of the obtained data for each target demonstrated linearity *R*^2^>0.99 for both pathogens withinthe range of 80 to 8,000,000 gene copies per reaction (Additional file 1: Fig. S1). The efficiency of the novel assay for *B. canis* was 103.1%, and that for *A. phagocytophilum* was 102.2%.

### Analytical sensitivity (LOD) of the RT-PCR assay

Analytical sensitivity for both targets (LOD) was tested by using 20 intra-assay replicates at two concentration levels: 5 and 10 gene copies per reaction. For both pathogens, ten gene copies per reaction resulted in 100% detection, while five gene copies per reaction were detected as positive only in 90% of cases for *B. canis* and 70% for *A. phagocytophilum* (Table 3). It was concluded that the LOD for the new RT-PCR method was ten gene copies per reaction for both pathogens.

**Table 3:** Lower limit of detection (LOD) of the RT-PCR assay for *B. canis* and *A. phagocytophilum* using 20 intra-assay replicates at two different concentration levels

Gene copies/ reaction	*B. canis Bc28* gene		*A. phagocytophilum mdh* gene	
	Mean CtSD	LOD, %	Mean CtSD	LOD, %
10	29.6291.058	(20/20) 100%	32.0730.997	(20/20) 100%
5	33.1971.044	(18/20) 90%	34.1410.522	(14/20) 70%

### Clinical specificity of the RT-PCR assay

The clinical specificity of the new RT-PCR assay was evaluated by analysis of two previously characterized clinical sample sets. In total, 351 clinical samples were tested, and the newly developed RT-PCR method demonstrated excellent accuracy, producing no false-positive or false-negative results (Table 4). *B. vulpes* (*n*=8)-, *E. canis* (*n*=3)- and *A. platys* (*n*=3)-positive samples did not show a positive signal (Additional file 2: Fig. S2). According to the PCR results, the novel RT-PCR assay provided correct classification of both *B. canis*- and *A. phagocytophilum*-positive and -negative samples.

**Table 4:** Characterisation of the sample sets used for clinical specificity assessment of the RT-PCR method

	*A. phagocytophilum-*positive	*B. canis*-positive	Pathogen-negative	Total	Correctly classified
Sample set no. 1	5	40	212	257	257
Sample set no. 2	1	2	91	94	94

## Discussion

Initially, for this study, we performed polymorphism analysis of the *Bc28.1* and *mdh* genes to determine the most suitable target regions for the novel RT-PCR method. The obtained results revealed sequence variability, which indicated the presence of genetically diverse *B. canis* and *A. phagocytophilum* isolates in canine clinical samples in Latvia. Interestingly, *B. canis* isolates belonging to the two main groups appeared in geographically separate locations, possibly indicating two separate events in the establishment of *B. canis* foci in Latvia. The predominant *Bc28.1* sequence type D197, which was mainly detected in the Riga and Riga regions, fully matched the previously reported *B. canis* A8 genotype (reference sequence CS019629.1 published in GenBank), whose prevalence in Europe was shown to increase from the south to center to north and from west to east [29]. This finding could provide novel data on *B. canis* expansion events in Europe. The other two sequence types have not been previously reported, while differences in *Bc28* sequences depending on geographical area were noticed earlier [29]. It would be interesting to explore this DNA marker in studies on piroplasmid diversity in other Northern European and Baltic countries and to determine whether these sequence types could be region specific. Additionally, two members of the *Bc28* gene family were characterized as major merozoite surface antigens playing a critical function in the interaction of merozoites with red blood cells [35]. Recently, Bc28 protein family members, which are secreted into the host bloodstream, were shown to be potential virulence factors [36]. However, it is still unknown whether the variability of the *Bc28* gene family could have any impact on the course of the disease or virulence of *B. canis*; future studies to address these questions are required. Additionally, several DNA markers are currently used for phylogenetic studies on *B. canis*; however, as efficiently highlighted by recent studies, the available sequence information is scarce, and more research is needed to identify highly specific and sensitive *B. canis* population variability markers [37, 38].

In parallel, the *A. phagocytophilum mdh* housekeeping gene, which encodes an enzyme catalyzing NAD+/NADH-dependent conversion of malate to oxaloacetate in various metabolic pathways, used in the MLST scheme designed for molecular characterization of *A. phagocytophilum*, was studied [39, 40]. Housekeeping genes are known to be highly conserved and have a low degree of variation. However, our sequence-based phylogenetic analysis of the *mdh* gene revealed that dogs were infected with two different *A. phagocytophilum* types belonging to the MLST database-based *mdh* gene clusters 2 and 3. Both *mdh* alleles 2 and 3 were described in different animals, including dogs, from numerous European countries and also were reported in humans. The presence of the widespread *A. phagocytophilum* strains in Latvian dogs is not surprising, and this finding could be of both veterinary and medical interest. Previous studies of the European *A. phagocytophilum* strains suggested the presence of different genetic variants and a correlation of these with the vertebrate hosts and tick vectors and also a possible correlation with geographical origin [41]. Thus, it would be important to explore the diversity of *A. phagocytophilum* in Latvia in more detail in future studies.

Based on the obtained results within this study, it was decided that the *Bc28.1* and *mdh* genes could be an appropriate and selective molecular targets of a novel RT-PCR method aiming for the detection of *B. canis* and *A. phagocytophilum* in clinical samples. At least seven *Bc28* gene copies are present in the *B. canis* genome, which probably could have a positive impact on the sensitivity of the molecular method [42]. Also, the low variability of the *mdh* gene could be considered a benefit for diagnostic purposes. The developed assay for simultaneous detection of both *A. phagocytophilum* and *B. canis* demonstrated high analytical sensitivity and was suitable for clinical samples. The LOD for both pathogens reached ten gene copies per reaction, and it was able to correctly classify positive and negative clinical samples. These results suggest that the *Bc28.1* and *mdh* genes appear to be good candidates for novel diagnostic strategies.

Duplex and multiplex RT-PCR tests could be beneficial in particular circumstances and settings such as when the risk of coinfection exists. Indeed, the presence of mixed infection of *B. canis* and *A. phagocytophilum* and triple infection with *Dirofilaria repens*, *A. phagocytophilum* and *B. canis* was detected in dogs in Slovakia [43, 44], and it was reported that outdoor dogs in Serbia are frequently exposed to the *Anaplasma* and *Babesia* genera [45]. *A. phagocytophilum* is widespread in Europe, while *B. canis* has been described across most of Europe with higher prevalence in central Europe [1, 10]. Coinfections with vector-borne pathogens may produce more severe clinical signs and alter clinical disease manifestations typically associated with singular infections thereby complicating diagnosis and treatment [46,49]. Thus, the duplex RT-PCR assay could provide suitable for simultaneous species-specific detection of *B. canis* and *A. phagocytophilum* infections in clinical samples and in screening studies of asymptomatic infections in dogs.

The limitation of this study was the inability to validate the RT-PCR method for the quantification of target gene copies in clinical samples, as the main purpose of the research was to develop and validate a qualitative method. In the future, it could be useful to upgrade this method and measure the exact quantity of pathogen DNA to evaluate the level of parasitemia/bacteremia.

## Conclusions

A novel duplex RT-PCR molecular method was developed for the detection of *B. canis* and *A. phagocytophilum* in canine clinical samples. Sequence variability of *Bc28.1* and *mdh* genes indicated the genetic variability of *B. canis* and *A. phagocytophilum* isolates occurring in Latvian domestic dogs.

## Supplementary Information

**Additional file 1: Figure S1.** Linearity of the novel duplex RT-PCR assay. Panel A: *B. canis*. Panel B: *A. phagocytophilum*.

**Additional file 2: Figure S2.** Specificity of the novel duplex RT-PCR assay. Representative results of the assay are shown. Abbreviations: *A. phagocytophilum*, *A. platys*, *B. canis*, *B. vulpes*, *E. canis*: pathogen-positive DNA samples. NK: negative control. Neg_DNA: pathogen-negative DNA sample. PK_BC28: recombinant plasmid standard control with *BC28.1* gene insert. PK_MDH: recombinant plasmid standard control with *mdh* gene insert.

## Data Availability

All data generated or analyzed during this study are included in this published article.

## Abbreviations

RT-PCR: Real-time polymerase chain reaction
MLST: Multilocus sequence typing
BLAST: Basic Logical Alignment Search Tool
LOD: Lower limit of detection
