# Supplementary figures and images for: Development of a real-time PCR method for rapid diagnosis of canine babesiosis and anaplasmosis
Source: Parasit Vectors. 2021 May 20;14:266. doi: 10.1186/s13071-021-04756-9 (PMC8139040; doi:10.1186/s13071-021-04756-9)

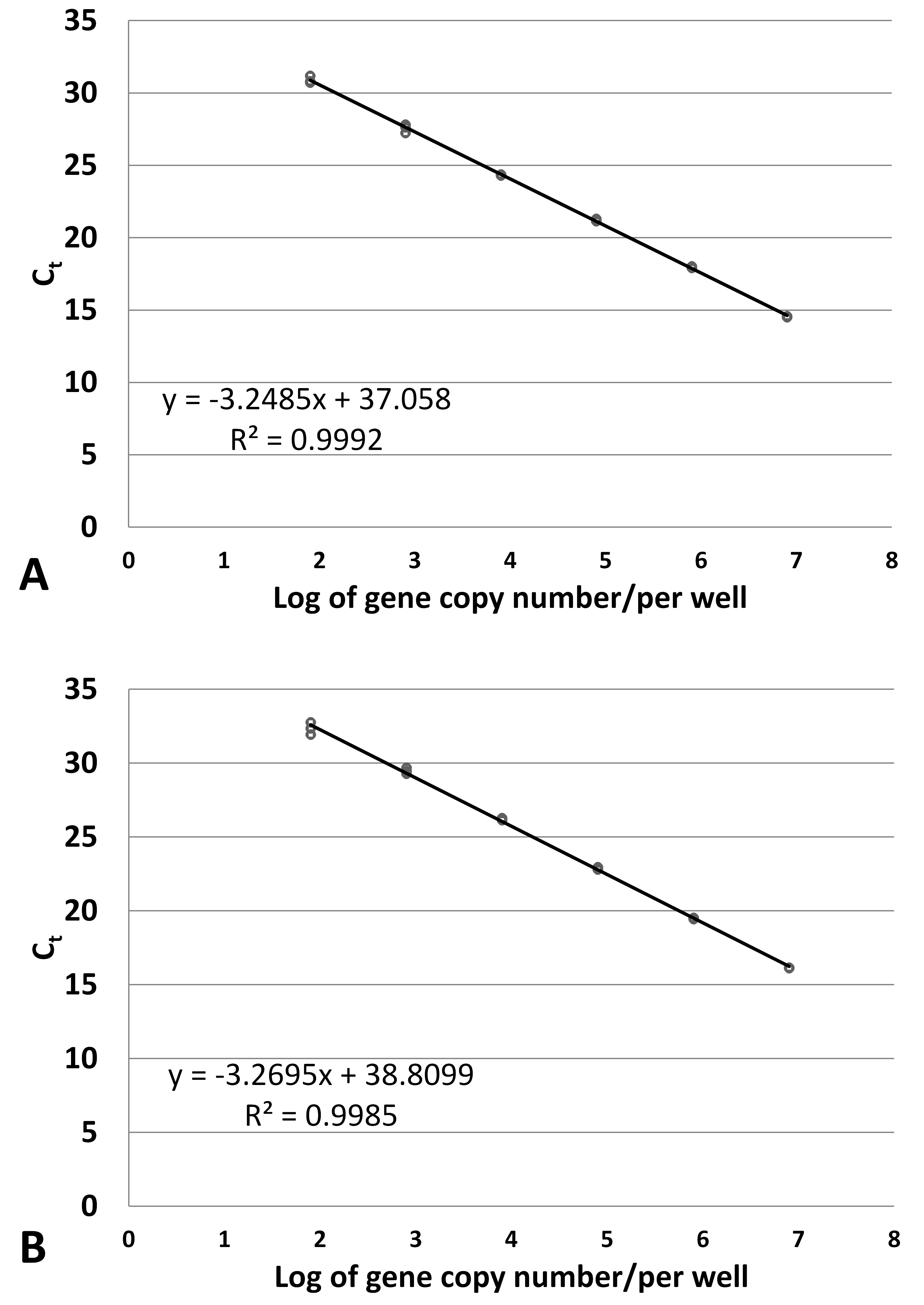

Supplement: Supplementary file 1 — Additional file 1: Figure S1. Linearity of the novel duplex RT-PCR assay. Panel A: B. canis. Panel B: A. phagocytophilum. [file 13071_2021_4756_MOESM1_ESM.tif]

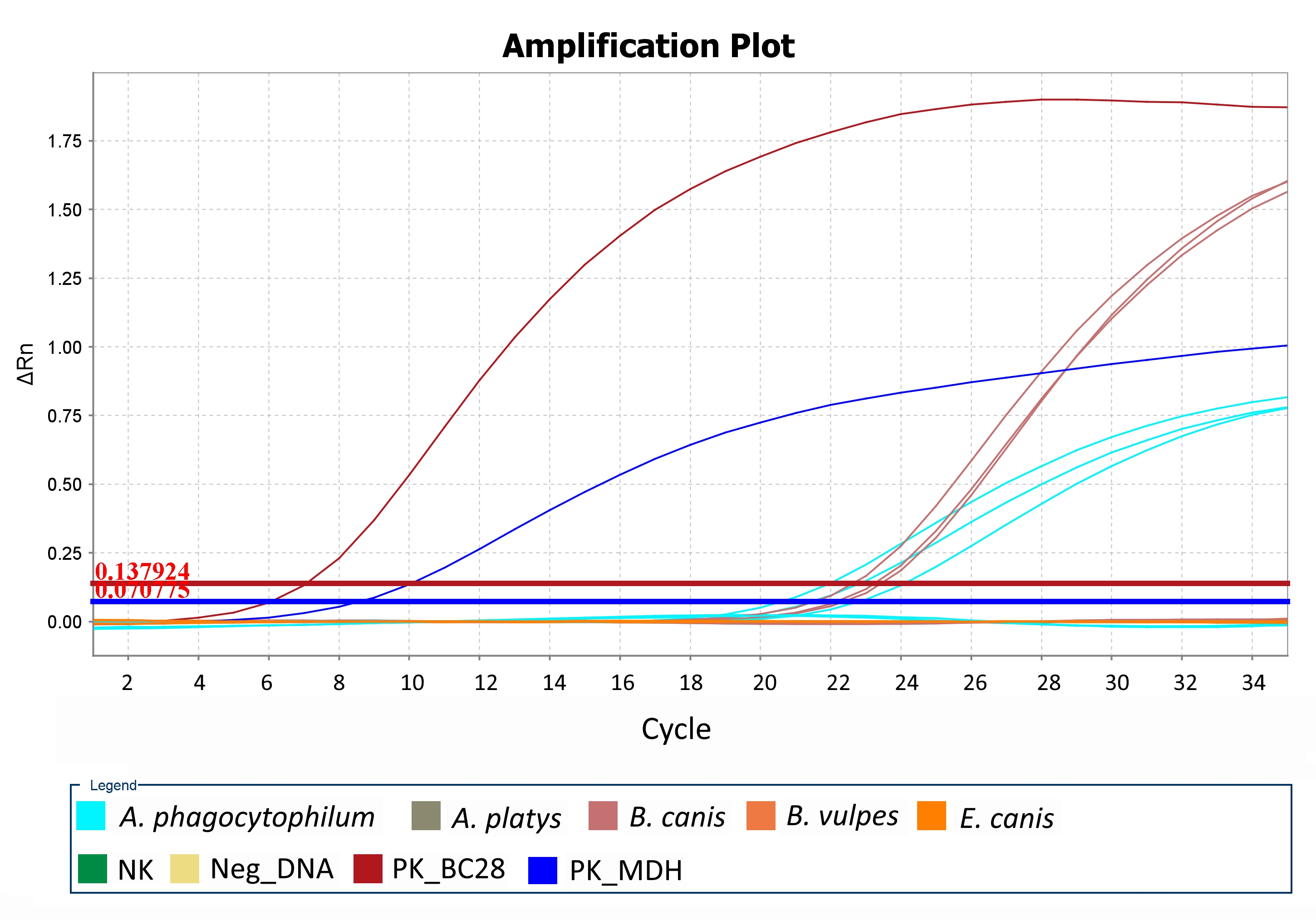

Supplement: Supplementary file 2 — Additional file 2: Figure S2. Specificity of the novel duplex RT-PCR assay. Representative results of the assay are shown. Abbreviations: A. phagocytophilum, A. platys, B. canis, B. vulpes, E. canis: pathogen-positive DNA samples. NK: negative control. Neg_DNA: pathogen-negative DNA sample. PK_BC28: recombinant plasmid standard control with BC28.1 gene insert. PK_MDH: recombinant plasmid standard control with mdh gene insert. [file 13071_2021_4756_MOESM2_ESM.tif]
